# Antineoplastic agents-associated hepatitis B virus reactivation: Research progress and molecular mechanisms

**DOI:** 10.1016/j.virusres.2025.199655

**Published:** 2025-11-01

**Authors:** Huajie Xie, Meijun Lv, Qin Jia, Yanyan Wang, Wanlin Na, Yuan Liu, Kai Chang

**Affiliations:** Department of Laboratory Medicine, The General Hospital of Western Theater Command, Chengdu, Sichuan Province, PR China

**Keywords:** HBV reactivation, ICIs, PD-1, PD-L1, CTLA-4

## Abstract

HBV infection is a major global health concern, leading to numerous HBV-related deaths. Due to the lack of curative or eradicative treatments for HBV, a large number of individuals in the general population are at risk of HBV reactivation. The increasing application of ICIs for treating various malignancies has raised concerns about HBV reactivation caused by these therapies. HBV reactivation complicates the treatments, leading to the interruption or modification of therapeutic regimens, which presents a considerable challenge. In this review, we summarize relevant researches on the definition, prevalence, and pathogenesis of HBV reactivation. Furthermore, we discuss the risks and mechanism of HBV reactivation during ICIs treatment, outline management strategies for HBV reactivation, and provide recommendations for assessing and monitoring HBV status during the treatment process.

## Highlights

• We summarized the recent research progress on hepatitis B virus reactivation.
• This article compares and analyzes existing mainstream guidelines for hepatitis B virus reactivation and presents viewpoints.
• This article discusses and analyzes the molecular mechanisms of hepatitis B virus reactivation.
• Based on the current status of hepatitis B virus reactivation research and treatment, this article puts forward recommendations.

## 1 Introduction

The hepatitis B virus (HBV), a small hepatropic DNA virus, has been infecting humans for thousands of years (Yardeni et al., 2023). HBV can cause liver damage through acute and chronic infection, with the majority of the disease burden arising from chronic infection's long-term consequences, primarily cirrhosis and hepatocellular carcinoma (HCC) (Perz et al., 2006; El-Serag, 2012). HBV infection is highly prevalent worldwide. According to the latest revelations of the World Health Organization's Global Hepatitis Report 2024, 254 million people were living with chronic HBV in 2022 (Easterbrook et al., 2024). In 2022, HBV caused approximately 1.1 million deaths (Easterbrook et al., 2024), primarily due to cirrhosis and HCC, representing an increase from 555,000 deaths in 2019 (Collaborators GBDHB, 2022). Viral hepatitis has become the second leading cause of infectious death globally.

Given the substantial burden on global public health, the WHO aims to eradicate HBV by 2030, targeting a 90 % reduction in incidence and a 65 % reduction in mortality compared to baseline levels in 2015 (Cox et al., 2020). However, as 2030 approaches, many countries are struggling to meet these elimination targets under the current frameworks (Polaris Observatory C, 2023). One significant challenge in managing HBV infection may be lie in that individuals ever infected with HBV are at risk of viral reactivation (Reddy et al., 2015). HBV reactivation typically involves a sudden rise in viral load or the reappearance of hepatitis B surface antigen (HBsAg) (G. Lau et al., 2021). The incidence of HBV reactivation ranges from 0 % to 100 %, depending on the HBV serological status of recipients, immunosuppressive therapies and antiviral prophylaxis regimens (G. Lau et al., 2021; Yin et al., 2023; Xia et al., 2023; Li et al., 2021; Myint et al., 2020). HBV reactivation can occur spontaneously, but it is often observed in the context of immunosuppression (G. Lau et al., 2021). In such scenarios, the immune system is further compromised, increasing the risk of HBV reactivation (Yin et al., 2023). The persistence of HCC risk with nucleoside analogue therapy (NUC) therapy, particularly when only started after the appearance of liver disease is also a significant problem. Recent modernization of treatment guidelines in China reflects this reality. Antiviral therapy should be initiated as soon as possible in patients with high risk of HCC and high viral load (≥8.00 log10 IU/mL).

The disappearance of HBsAg from the serum, the development of anti-HBs antibodies, and normalization of liver function may not reflect complete virological recovery from acute HBV infection. HBV DNA, and possibly virions, can remain detectable in the serum, with the viral genome persisting in a transcriptionally active form in PBMCs for over five years after complete clinical and serological recovery from acute viral hepatitis (Michalak et al., 1994; Rehermann et al., 1996).

Immune checkpoint inhibitors (ICIs) work by blocking checkpoint proteins from binding with their partner proteins. This prevents the “off” signal from being sent, allowing the T cells to kill cancer cells. Recently, the growing application of ICIs such as programmed cell death protein 1/programmed cell death 1 ligand 1 (PD-1/PD-L1) and cytotoxic T-lymphocyte-associated antigen 4 (CTLA-4) inhibitors in treating various cancers has brought attention to HBV reactivation due to the immune balance disruption caused by these therapies (Shiravand et al., 2022; Lasagna et al., 2021; Godbert et al., 2021). Many studies highlight the landscape of HBV reactivation in patients undergoing immunotherapy with ICIs (Ding et al., 2023). In these patients, HBV reactivation complicates treatment, often leading to interruptions or modifications in therapeutic regimens, which poses significant challenges. In this review, we summarize relevant research on the definition, prevalence, and pathogenesis of HBV reactivation. Additionally, we examine the risks and mechanisms of HBV reactivation during ICI treatment, outline management strategies, and offer recommendations for assessing and monitoring HBV status during such therapies.

## 2 Definition

Broadly, HBV reactivation involves significant disruptions in the balance between viral replication and host immune control (Chang et al., 2021). For HBsAg carriers, it is defined as an increase in HBV DNA levels compared to baseline (Myint et al., 2020). In HBsAg-negative but anti-hepatitis B core (HBc)-positive patients with undetectable HBV DNA, reactivation is defined by either reverse seroconversion of HBsAg or detectable serum HBV DNA (Huang et al., 2020). While these criteria are widely accepted, the specific cutoff value for HBV DNA levels that define reactivation are different among studies and guidelines. The guidelines from the American Association for the Study of Liver Diseases (AASLD) define HBV reactivation as: 1) for HBsAg-positive, anti-HBc–positive patients: a 100-fold increase in HBV DNA levels compared to baseline, ≥1000 IU/mL in patients with previously undetectable levels, or ≥10,000 IU/mL if baseline levels are unavailable; 2) for HBsAg-negative, anti-HBc-positive patients: HBV DNA is detectable or reappearance of HBsAg (Terrault et al., 2018). The Asian Pacific Association for the Study of the Liver (APASL) guidelines describe reactivation as a 100-fold increase from baseline, or new detection of HBV DNA at ≥100 or ≥20,000 IU/mL in those without baseline levels (Sarin et al., 2016). The guideline of the European Association for the Study of the Liver (EASL) does not specify an absolute value for HBV DNA increase to define reactivation (European Association for the Study of the Liver, 2017). The Chinese Medical Association (CMA) guidelines describe reactivation as HBV DNA increase ≥ 2log10 IU/mL in patients with a stable HBV DNA levels, or new detection of HBV DNA at ≥100 or ≥20,000 IU/mL in those without baseline levels (Table 1). While the appearance HBV DNA at the thresholds for indicated by various guidelines does indeed reflect technical reactivation, it is important to discern when current guidelines recommend implementation of treatment based on prognosis. For instance, a patient reactivating their HBV infection with HBV DNA < 2000 IU/mL with no evidence of liver disease would not be a candidate for therapy since these patients have a very low risk for developing liver disease (G. Lau et al., 2021).

**Table 1:** Definition of HBV reactivation in different regions.

Guidelines Institution	Previous state	Definitions
AASLD	HBsAg-positive, anti-HBc–positive	a 100-fold increase in HBV DNA levels compared to baseline, ≥1000 IU/mL in patients with previously undetectable levels, or ≥10,000 IU/mL if baseline levels are unavailable
	HBsAg-negative, anti-HBc-positive	HBV DNA is detectable or reappearance of HBsAg
APASL		a 100-fold increase from baseline, or new detection of HBV DNA at ≥100 or ≥20,000 IU/mL in those without baseline levels
EASL		specify an absolute value for HBV DNA increase to define reactivation
CMA		HBV DNA increase ≥ 2log10 IU/mL in patients with a stable HBV DNA levels, or new detection of HBV DNA at ≥100 or ≥20,000 IU/mL in those without baseline levels.

Note: AASLD: American Association for the Study of Liver Diseases; APASL: Asian Pacific Association for the Study of the Liver; EASL: European Association for the Study of the Liver; CMA: Chinese Medical Association.

The absence of a standardized, cross-disciplinary definition for HBV reactivation has become a critical bottleneck limiting both scientific progress and clinical standardization in this field. In comparative studies, the use of varying viral load thresholds leads to high heterogeneity, hindering systematic evaluation of risk factors or mechanistic associations through meta-analyses and weakening the cumulative value of scientific evidence. Meanwhile the ambiguity of definitions exacerbates uncertainty in clinical decision-making: discrepancies in criteria may lead to delayed interventions or overtreatment, ultimately affecting patient outcomes and increasing healthcare costs. Moreover, the lack of a unified basis impedes the development of authoritative clinical guidelines, limiting the coordination of global prevention strategies. Therefore, interdisciplinary collaboration is urgently needed to establish a standardized definitional framework based on pathophysiological features and clinical endpoints (e.g., virological response, liver injury grading), in order to bridge the gap between research and clinical practice, ensure patient safety, and optimize treatment strategies.

## 3 Prevalence

The expanding application of immunosuppressive and immunomodulatory therapies across diverse disciplines—including oncology, rheumatology, gastroenterology, neurology, and dermatology—has significantly heightened the risk of hepatitis B virus (HBV) reactivation in patients with overt or occult HBV infection (Loomba and Liang, 2017). Current evidence demonstrates that the incidence of HBV reactivation varies substantially depending on the HBV serological status of recipients, immunosuppressive therapies and antiviral prophylaxis regimens. Notably, detectable HBV DNA levels and specific serological markers such as HBsAg, anti-HBc, and hepatitis B e-antigen (HBeAg) have been identified as critical predictors of reactivation risk (Pattullo, 2016). A study on these risk factors found that 37.8 % of participants with detectable HBV DNA levels experienced reactivation, highlighting HBV DNA as a significant risk factor (Yeo et al., 2004). Patients with detectable HBsAg face up to an 8-fold increased risk of HBV reactivation compared to those who are HBsAg-negative/anti-HBc-positive (Shouval and Shibolet, 2013). Notably, patients with undetectable HBV DNA at baseline are still at risk for HBV reactivation (Yeh et al., 2020). A recent study has shown that baseline quantitative HBsAg (qHBsAg) titers correlated with reactivation; a 1-year cumulative incidence rate of 42.5 % was observed in patients with baseline qHBsAg >10 IU/mL, compared to 18.5 % in those with lower levels (Yeh et al., 2020). HBsAg possesses well-documented immunosuppressive properties, and the risk of reactivation is correlated with its levels in the blood, regardless of mutation status (Vaillant, 2020). Additionally, mutations in HBsAg may impair humoral response, increasing the risk of HBV reactivation (Pattullo, 2016). Moreover, HBeAg-positive patients are more prone to reactivation compared to HBeAg-negative patients (Yeo et al., 2004). Notably, existing studies suggest that HBV genotype may also influence HBV reactivation, but further studies are required to confirm this association (Pattullo, 2016; Mozessohn et al., 2015).

Immunosuppression from any cause can impair immune-mediated control of HBV replication, potentially leading to the reactivation (Chang et al., 2021). Various immunosuppressive therapies, including chemotherapeutics, targeted therapies, interventional therapies, cell-based therapies, can make a patient susceptible to HBV reactivation. Notably, novel immunomodulatory agents such as immune checkpoint inhibitors (ICIs) can lead to immune dysfunctions and suppress anti-HBV immunity (Shi and Zheng, 2020). HBsAg-positive breast cancer patients receiving chemotherapy had an HBV reactivation risk of about 22 % (range from 14 to 41 %), and the risk of HBV-related hepatitis flare, defined as an event of abrupt ALT elevation to >5 × upper limit of norma (ULN), was around 11 % (range from 0 to 21 %) (G. Lau et al., 2021). In lymphoma patients who were treated with rituximab-containing chemotherapy, 25 % developed HBV reactivation and 20 % died of hepatic decompensation (Yeo et al., 2009). Tyrosine kinase inhibitors (TKIs), commonly used for lung cancer and chronic myeloid leukemia (CML), were associated with a 26–38.5 % risk of HBV reactivation among CML patients receiving imatinib (G. Lau et al., 2021; Y.H. Wang et al., 2019). The HBV reactivation risk for HBsAg-positive cancer patients on steroid-containing regimens was 26–72 %, compared to 13–36 % for those on non-steroid regimens (Cheng et al., 2003). In HBV-related HCC patients undergoing transarterial chemoembolization, the study has shown a 30 % reactivation risk (Jang et al., 2006; Jang et al., 2011). A 45–100 % risk of HBV reactivation and 15 % hepatic failure were observed in HBsAg-positive patients receiving hematopoietic stem cell transplantation (Dore et al., 2010; Nakamoto et al., 2014). In a study of 19 patients with B-cell malignancies and chronic HBV infection, one patient (5.3 %) experienced HBV reactivation and severe HBV-related hepatitis following anti-CD19 chimeric antigen receptor (CAR)-T-cell therapy, despite the treatment with entecavir (Cao et al., 2020). Two additional studies on patients with B-cell or plasma-cell malignancies and chronic HBV infection reported HBV reactivation after CAR-T-cell therapy in 3 of 15 patients (20 %) in one study and 2 of 12 patients (16.7 %) in the other (Yang et al., 2020; Wang et al., 2020). In terms of patients receiving ICIs therapy, the incidence of HBV reactivation also varied greatly across different studies (Xia et al., 2023), which will be described in detail below. Antiviral prophylaxis had been reported to significantly reduce the risk of HBV reactivation (Su et al., 2018; Cheung et al., 2020). Among the cancer patients undergoing chemotherapy, the incidence of HBV reactivation has been reported to be 0 %−38 % in patients receiving non-antiviral prophylaxis and 0 % to 7 % in patients receiving antiviral prophylaxis (Wu et al., 2017). This substantial variability is attributable to several confounding factors not uniformly adjusted for across studies. Key sources of heterogeneity include patient baseline characteristics, geographic epidemiology variations, chemotherapy regimens and prophylactic strategies. Consequently, a systematic consideration of these factors is imperative when interpreting these widely variable reactivation rates and deriving generalizable clinical conclusions. Future research and clinical practice should aim for refined risk stratification, integrating these variables to inform more personalized management strategies.

## 4 Pathogenesis

**Fig. 1:**
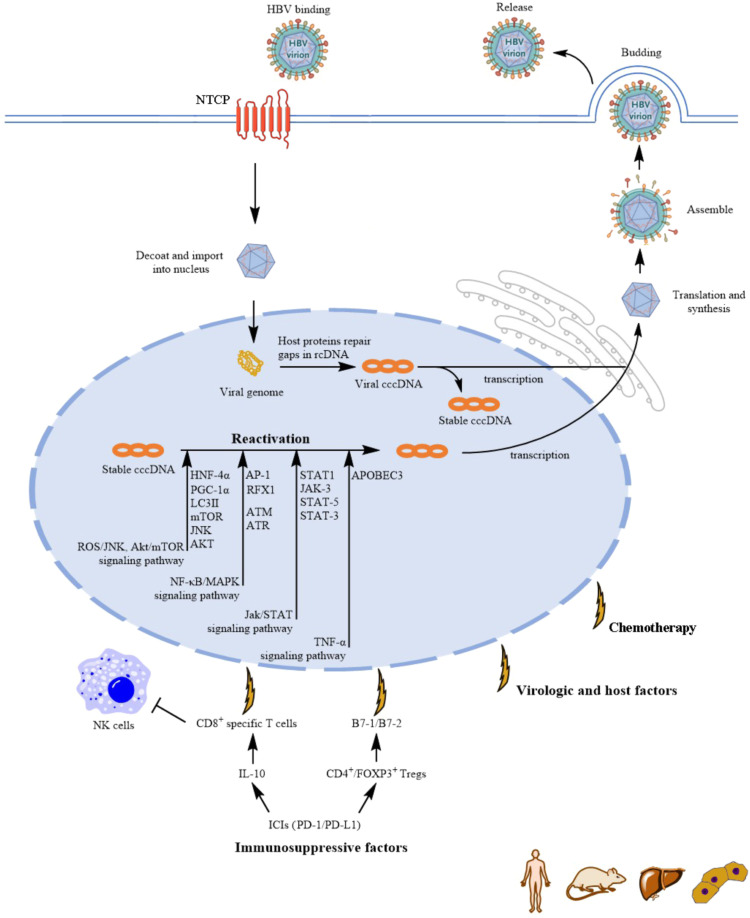
Mechanisms of HBV reactivation. This illustration depicts the complete HBV life cycle from hepatocyte infection to viral replication, the key virologic and host factors involved in Hepatitis B Virus (HBV) reactivation, from viral entry and persistence to immune regulation and signaling pathways, also highlighting the role of immune checkpoints in this process. rcDNA: Relaxed Circular DNA; cccDNA: Covalently Closed Circular DNA; HNF-4α: Hepatocyte Nuclear Factor 4 Alpha; AP-1: Activator Protein 1; APOBEC3: Apolipoprotein B mRNA Editing Enzyme Catalytic Polypeptide-like 3; PGC-1α: Peroxisome Proliferator-Activated Receptor Gamma Coactivator 1-alpha; ROS: Reactive Oxygen Species; JNK: c-Jun N-terminal Kinase; Akt: Protein Kinase B (also known as PKB); mTOR: Mammalian Target of Rapamycin; LC3II: Microtubule-Associated Protein 1A/1B-Light Chain 3-II; TNF-α: Tumor Necrosis Factor Alpha; NF-κB: Nuclear Factor Kappa B; MAPK: Mitogen-Activated Protein Kinase; JAK: Janus Kinase; STAT: Signal Transducer and Activator of Transcription; ATM: Ataxia Telangiectasia Mutated; ATR: ATM and Rad3-Related; FOXP3: Forkhead Box P3; ICIs: Immune Checkpoint Inhibitors; PD-1: Programmed Cell Death Protein 1; PD-L1: Programmed Death-Ligand 1.

HBV reactivation can occur spontaneously due to virologic and host factors, but it is commonly triggered by immunosuppressive factors (Smalls et al., 2019), causing acute or chronic infections (Chang et al., 2021). Once entering hepatocytes, HBV releases its partially double stranded viral genome, which is then imported to the nucleus (Fig. 1) (Marchetti et al., 2022). Here, the incoming relaxed circular DNA (rcDNA) is repaired by host nuclear proteins into covalently closed circular DNA (cccDNA) (Newbold et al., 1995), which is the key to HBV persistence. The cccDNA is comprised of histones as well as nonhistone host proteins, acquires a chromatin-like structure in the nucleus and assembles into a chromatinized episome termed minichromosome, Which is the major template for HBV gene transcription (Marchetti et al., 2022; Gómez-Moreno and Ploss, 2024). It is transcriptionally active and a few copies of cccDNA can drive significant amount of progeny virus production in vivo and in culture. Even with transcriptionally silent, the cccDNA remains in hepatocytes in a latent state, acting as a reservoir for potential HBV reactivation (Chang et al., 2022). Due to the persistent existence of cccDNA, HBV reactivation can occur whenever the balance between the host immune system and viral replication is disrupted (Chang et al., 2021; Smalls et al., 2019). When decondensed and transcriptionally active, cccDNA supports the production of new viral and subviral particles (Jin et al., 2023). Subsequently, the immune response to the markedly increased HBV antigen expression results in liver injury, manifested as hepatitis and fulminant hepatic failure (G. Lau et al., 2021).

Clinical recovery from HBV infection does not indicate a complete cure of HBV infection, as cccDNA exists persistently in hepatocyte nuclei (Werle-Lapostolle et al., 2004). Theoretically, a complete cure of HBV infection requires the eradication of HBV cccDNA, which would make viral replication impossible (Lok et al., 2017). However, current host immune responses and antiviral treatments with NUC therapy cannot fully eliminate both HBV cccDNA and integrated DNA (Chang et al., 2021). HBV reactivation occurs based on the fully replication-competent cccDNA remains in the nuclei of infected hepatocytes (Chang et al., 2021). Even if only one copy of cccDNA remains, HBV replication can cause detectable viremia upon reactivation (Chang et al., 2021).

Based on the persistence of cccDNA, HBV reactivation may occur under many circumstances. In terms of the potential mechanisms underlying chemotherapeutic-related HBV reactivation, endoplasmic reticulum (ER) stress-peroxisome proliferator-activated receptor gamma coactivator 1 alpha (PGC-1α) signaling pathway may play a critical role in cisplatin-induced HBV reactivation (Table 1) (Li et al., 2018). In this context, cisplatin treatment leads to an increase in the expression levels of PGC-1α and hepatocyte nuclear factor 4 alpha (HNF-4α). PGC-1α then coactivates with HNF-4α, which binds to the core promoter and enhancer II region of the HBV genome, consequently enhancing HBV production (Li et al., 2018). Moreover, cisplatin was reported to induce autophagy via the activation of ROS/JNK pathway and the inhibition of the Akt/mTOR pathway, which in turn enhanced HBV replication (Chen et al., 2019). Paclitaxel may trigger HBV reactivation as a synergistic result of immunosuppression and direct stimulation of HBV replication by enhancing HBV core promoter activity (Chen et al., 2024). Transcriptome sequencing has shown that paclitaxel activates immune-related signaling networks, including IL-17, Nuclear Factor kappa B (NF-κB), and mitogen-activated protein kinase (MAPK) pathways. The critical common molecule in these pathways is the transcription factor AP-1 (Chen et al., 2024). Doxorubicin, another cytotoxic chemotherapy drug, was found to promote HBV replication via regulatory factor X box 1 gene (RFX1) and HBV enhancer I, which may participate in doxorubicin‐induced HBV reactivation (Wang et al., 2018). At the same time, some studies indicate that DNA damage caused by Doxorubicin and H2O2 significantly elevates HBV replication and HBV reactivation, which may be due to transcriptional activation of ataxia-telangiectasia mutated (ATM) and ATM- and RAD3-related (ATR) in response to DNA damage (Kostyusheva et al., 2019). The downregulation of transcription of ATR and ATM reduced HBV transcription, and intracellular and secreted HBV DNA levels by 50–80 %. Recent clinical reports have highlighted HBV reactivation associated with Janus kinase (JAK) inhibitors (Pan et al., 2023). The variable responses of JAK inhibitors regarding HBV reactivation risks are not well understood. A potential mechanism for HBV reactivation induced by JAK inhibitors could involve the JAK-STAT pathway (Pan et al., 2023), which is critical for immune signal transduction, cellular development, growth, and apoptosis, exhibiting significant immunomodulatory effects (McLornan et al., 2015). JAK inhibitors might lead to increased HBV reactivation risk by impairing functions of dendritic cells and T cells, reducing populations of circulating natural killer cells, and limiting the antiviral actions of interferon (Pan et al., 2023). TNF-α has the ability to trigger a distinct antiviral mechanism in the host, the APOBEC (apolipoprotein B mRNA editing enzyme, catalytic polypeptide-like) proteins, which results in the degradation of cccDNA within cells infected by HBV (Lucifora et al., 2014). Consequently, inhibitors of TNF-α could facilitate the replication and reactivation of HBV by inhibiting this specific TNF-α-driven antiviral signaling pathway (Chang et al., 2021). TKIs may disrupt lymphocyte function and simultaneously trigger HBV reactivation by blocking TKI-mediated signaling pathways, which are crucial for immune activation and lymphocytes proliferation (B. Wang et al., 2019; Lee et al., 2020).In addition, TKIs such as imatinib mesylate, nilotinib, dasatinib were found to inhibit the phosphorylation of Bruton's tyrosine kinase (Btk) and its downstream substrates phospholipase C-γ2 (PLC-γ2) in a dose-dependent manner, which disrupts B lymphocyte activation and induction of humoral immune response to trigger HBV reactivation through off-target multikinases inhibitory effects (de Lavallade et al., 2013).Treatment with mammalian target of rapamycin (mTOR) inhibitors can enhance HBsAg synthesis at the transcriptional level through a feedback mechanism (Teng et al., 2011), which might lead to increased HBV replication and reactivation. Moreover, it was reported that radiotherapy combined with IL-6 was able to induce HBV reactivation, mainly mediated through the STAT3 signaling pathway and involving the cooperative interaction of p-STAT3/HNF-3 protein complex with the HBV enhancer 1 (Chou et al., 2009).

Taken together, some progress has been made in elucidating the complex immunological mechanism behind the reactivation of HBV, but a comprehensive understanding of its pathophysiology remains elusive due to the limited evidence. Therefore, further extensive research is essential to explore the mechanism that govern the transition from controlled viral replication to reactivation, and how this impacts immune responses.

## 5 HBV reactivation during ICIs

Given the substantial number of HBV carriers, it is common for many cancer patients to have a concurrent HBV infection (Xia et al., 2023). ICIs have revolutionized the cancer therapies, and the population qualifying for ICIs therapy is expanding (Sharma et al., 2023). HBV reactivation in cancer patients with concurrent HBV infection following ICIs treatment has attracted much attentions. ICIs have the potential to restore virus-specific T-cell responses and inhibit HBV replication theoretically (Wykes and Lewin, 2017; Zhao et al., 2022). Intriguingly, accumulating data suggest that ICIs may induce HBV reactivation. Here, we review pertinent studies on the risk and mechanisms of HBV reactivation during ICIs treatment and discuss strategies for managing HBV reactivation.

### 5.1 Risks

HBV reactivation can occur in patients with chronic hepatitis B (HBsAg-positive) as well as in those with resolved HBV infection (HBsAg-negative/HBcAb-positive) during immunotherapy, potentially leading to severe complications for cancer patients (Papatheodoridi et al., 2022; Papatheodoridis et al., 2022). Moreover, HBV reactivation may result in the interruption of antineoplastic therapy, thereby affecting overall survival rates (Shen et al., 2023).

A recent meta-analysis of 34 studies involving a combined cohort of 7126 patients assessed the incidence of HBV reactivation in patients undergoing ICI-based therapy for advanced cancer (Xia et al., 2023). The findings indicated that the prevalence of HBV reactivation varied widely across studies, ranging from 0 % to 30.05 % (Xia et al., 2023). Patients diagnosed with HCC, HBV carriers, and individuals from Asian regions or developing countries experience higher rates of HBV reactivation (Xia et al., 2023). A retrospective study of 180 HBsAg-positive cancer patients treated with PD-1 inhibitors reported HBV reactivation in 11 patients (6.11 %) and immune-related adverse events (irAEs) in 36 patients (20 %); and the multivariable analysis s identified irAEs as the sole independent risk factor for HBV reactivation (OR: 5.56, (Chou et al., 2009), *P* = 0.01) (Zeng et al., 2024). In HCC patients receiving a combination of PD-1 inhibitors, angiogenesis inhibitors, and concurrent first-line antivirals, 16 out of 218 patients (7.3 %) experienced HBV reactivation (Wang et al., 2024). Hepatitis B e antigen (HBeAg) positive patients undergoing combination therapy had a 17-fold higher risk of HBV reactivation compared to HBeAg-negative patients receiving PD-1 inhibitor monotherapy (Wang et al., 2024). Patients treated with a combination of PD-1 inhibitors and TKIs for advanced HCC had a higher risk of HBV reactivation compared to those receiving TKIs alone, with 3-, 6-, and 12-month cumulative incidence rates of HBV reactivation being 9.9 %, 19.2 %, and 30.0 % versus 7.8 %, 12.8 %, and 21.3 % (Lei et al., 2023). Furthermore, patients experiencing HBV reactivation exhibited higher rates of tumor progression and shorter survival times than those treated with TKIs alone (Lei et al., 2023). Even with concurrent antiviral prophylaxis during PD-1 inhibitor treatment, patients with high HBV-DNA loads (>500 IU/ml) were more prone to HBV reactivation compared to those with low HBV-DNA loads (5.3 % vs 1.9 %) (He et al., 2021). However, during ICIs therapy, the risk of HBV reactivation was significantly higher in chronic HBV patients not receiving antiviral prophylaxis compared to those who were (17 % vs 1 %, *p* < 0.05) (Mustafayev et al., 2024). In a real-world setting, HBV reactivation was observed in 14.3 % (2 out of 14) of HBsAg-positive NSCLC patients who were not on antiviral therapy (Wang et al., 2024).

Taken together, ICIs, whether used as monotherapy or in combination with other agents, can lead to HBV reactivation, particularly in patients with chronic HBV infection who are not receiving antiviral prophylaxis. To effectively identify at-risk patients and administer prophylactic antiviral agents to prevent HBV reactivation, a deep understanding of the mechanisms by which ICIs trigger HBV reactivation is essential.

### 5.2 Mechanisms

ICIs are employed in the treatment of a wide range of solid cancers by restoring the anti-tumor functions of T cells (Sharma et al., 2023). It appears plausible that inducing functional HBV-specific T cells could be an effective strategy for HBV clearance, given that virus-specific T cells can eliminate cccDNA-carrying cells in approximately 90 % of infected patients (Zhang et al., 2015). However, this outcome is unexpected because ICIs overcome T cell exhaustion, and subsequently impairs the control of viral replication (Shi and Zheng, 2020).

Regulatory T cells (Tregs), a T-cell subpopulation capable of suppressing the activation of CD4+ and CD8+ *T* cells, highly express cytotoxic T-lymphocyte-associated antigen 4 (CTLA-4) on their surface. Therefore, blocking CTLA-4 may activate Tregs and consequently impair the ability of T cells to maintain suppression of the HBV. A small-scale clinical study indicated that the CTLA-4 inhibitor tremelimumab increases the number of Tregs in peripheral blood (Sangro et al., 2013). Meanwhile, specific CD8+ *T* cells play a central role in controlling HBV infection by recognizing and eliminating infected cells. The persistence of hepatitis B virus leads to CD8+ *T* cell exhaustion through negative co-stimulatory molecules such as CTLA-4 and PD-1. Blocking these molecules can reverse this T-cell exhaustion and thereby enhance the antiviral response. However, these effects may disrupt the balance between the host immune system and viral control, consequently increasing the risk of HBV reactivation and liver function damage (Nishijima et al., 2017).

Several potential mechanisms (Table 2) have been proposed to explain the reactivation of HBV following ICIs therapy, though none has been definitively confirmed An attractive hypothesis proposes that ICIs may reverse T cell exhaustion and restore antiviral CD8⁺ T cell function in chronic viral hepatitis. Under chronic viremia, sustained overexpression of PD-1 on hepatocytes drives T cell exhaustion. Upon binding to its ligands PD-L1/PD-L2, PD-1 recruits phosphatases such as SHP-1/2, leading to the dephosphorylation and inhibition of critical signaling molecules in the T cell receptor pathway—including ZAP70 and CD3δ. This suppression attenuates downstream Akt and mTOR pathway activation, thereby promoting T cell apoptosis (Jiang et al., 2019). Blocking the PD-1/PD-L1 axis interrupts this inhibitory signaling, restoring the T cell receptor (TCR) signal fidelity and intensity, which in turn reverses functional exhaustion of HBV-specific CD8⁺ T cells and restores their proliferative capacity and enhances the production of effector cytokines such as IFN-γ and TNF-α (Hofmeyer et al., 2011).However, this potent immune reactivation represents a double-edged sword: while facilitating viral clearance, it may also induce severe liver injury and provoke the release of previously latent virus into the bloodstream (Ziogas et al., 2020; Knolle and Thimme, 2014). Moreover, PD-1 blockade may facilitate the proliferation of Cluster of Differentiation 4 (CD4^+^)/forkhead box P3 (FOXP3^+^) regulatory T cells (Tregs), which downregulate the expression of B7–1/B7–2 on liver sinusoidal endothelial cells, limiting the ability of antigen-presenting cells to activate CD4^+^ *T* cells, thereby increasing immunosuppression and then inducing HBV reactivation (Li et al., 2020; Kamada et al., 2019). Meanwhile, PD-1 blockade may skew the effector T cells (Teff) /Treg balance toward Tregs. This shift is mediated by anti-PD-1-induced activation of the TGF-β/Smad3 pathway within tumor cells. Subsequent Smad3 upregulation potentially drives epithelial-to-mesenchymal transition (EMT) towards myofibroblast phenotype, diminishes antigen presentation, and ultimately compromises immune surveillance (Dodagatta-Marri et al., 2019). Furthermore, PD-1 blockade may promote the proliferation of myeloid-derived suppressor cells (MDSCs) (Strauss et al., 2020), which could regulate the immune response in chronic HBV- infected patients via PD-1 induced IL-10; and they may also promote the induction of Tregs and exhausted CD8^+^ *T* cells and inhibit NK cells in HCC (Li et al., 2020). MDSC level is considered as a new biomarker for immune dysfunction, such as irAEs (Strauss et al., 2021). Additionally, inflammatory Treg reprogramming is suggested a characteristic of irAEs (Grigoriou et al., 2021). These findings may explain why the occurrence of irAEs is a risk factor for HBV reactivation (Zeng et al., 2024).

**Table 2:** Mechanisms of HBV reactivation.

Inducer	Signaling Pathway	Gene	Study object	First author Reference
Cisplatin	ROS/JNK, Akt/mTOR	LC3Ⅱ JNK/AKT	HepAD38	Chen (Chen et al., 2019)
Cisplatin		PGC-1α/HNF-4α	Vivo mice/ HepG2 cells	Li (Li et al., 2018)
Paclitaxel	IL-17/NF-κB/MAPK	AP-1	Vivo mice/ HepAD38	Chen (Chen et al., 2024)
Doxorubicin		RFX1	HepG2 cell	Wang (Wang et al., 2018)
Doxorubicin		ATM/ATR	HepG2 cell	Kostyusheva (Kostyusheva et al., 2019; Rowshanravan et al., 2018)
JAK inhibitor	JAK/STAT			Pan (Pan et al., 2023)
TNF-α inhibitor	TNF-α	APOBEC3	dHepaRG cells	Lucifora (Lucifora et al., 2014)
TKI	BCR signaling	Btk/phospholipase C-γ2		de Lavallade H (de Lavallade et al., 2013)
mTOR inhibitor	mTOR	mTOR/ pre-S1 promoter	HuH-7 cell	Teng (Teng et al., 2011)
Radiotherapy	STAT	STAT3/IL6	Vivo mice	Chou (Chou et al., 2009)
PD-L1 Blocked	STAT	JAK-3/STAT-5	ex-vivo PBLs or IHLs	Franceschini (Franceschini et al., 2009)

Similar to PD-1/PD-L1 axis, CTLA 4 inhibits T cell proliferation and survival, as well as reducing cytokine production. CTLA-4 blockade primarily suppresses the initial activation of T cells in lymph nodes, a process potentiated by Treg-mediated transendocytosis of B7 molecules from antigen-presenting cells (APCs) (Rowshanravan et al., 2018; Qureshi et al., 2011). In contrast, PD-1 blockade mostly influences the effector stage of the immune response in peripheral tissues (Ahmadzadeh et al., 2025).

### 5.3 Treatment strategies

Several professional societies in the United States and Europe recommend HBV prophylaxis for cancer patients with chronic HBV infection who are receiving ICIs because of its proven effectiveness in preventing HBV reactivation (Terrault et al., 2018; European Association for the Study of the Liver, 2017; Papatheodoridis et al., 2022; Hwang et al., 2020). Chronic HBV infection should not be considered a contraindication for ICI therapy (Mustafayev et al., 2024). It is essential to screen cancer patients for HBV infection before initiating ICIs therapies and to monitor HBV markers, such as HBsAg and HBV DNA, during the course of ICI treatment (Mustafayev et al., 2024; Hwang et al., 2020). Current international consensus recommends comprehensive risk stratification for all patients based on serological markers: systematic testing for HBsAg, anti-HBc, and anti-HBs must be completed before treatment initiation to accurately identify the three risk groups—chronic infection, past infection, and susceptible populations (Schneider et al., 2025).

Differentiated management strategies should be adopted for distinct risk groups. For HBsAg-positive patients, high-efficacy antiviral prophylaxis with agents such as entecavir or tenofovir should be initiated concurrently with the first ICI administration, regardless of baseline viral load—a strategy explicitly endorsed by the European Society for Medical Oncology guidelines (Haanen et al., 2025). For serological evidence of past infection (HBsAg-negative/anti-HBc-positive), particularly in anti-HBs-negative individuals or those receiving combined immunotherapy, prophylactic antiviral therapy is recommended. If a monitoring strategy is selected, a rigorous surveillance system with HBV DNA testing every 4–6 weeks must be established (Haanen et al., 2025).

Considering the potential for persistent immunological alterations induced by ICIs, the duration of antiviral therapy should adequately cover the risk window. It is recommended to continue antiviral prophylaxis for at least 6 months after ICI discontinuation, extending to 12 months for patients who have received potent immunosuppression. Following prophylaxis cessation, HBV DNA monitoring should be maintained for no <12 months. This long-term management strategy fully accounts for the unique sustained immune effects of ICIs (Terrault et al., 2018).

Currently, the treatment of chronic hepatitis B aims to achieve a "functional cure," defined as the clearance of HBsAg. However, current standard therapies—nucleos (t)ide analogs (NAs) and pegylated interferon (PEG-IFNα)—though effective in suppressing viral replication, rarely accomplish this ultimate goal, with HBsAg clearance rates remaining very low (Sugumar and Rengarajan, 2025). The underlying challenge lies in the fact that the clinical outcome of HBV infection heavily relies on a complex interaction between the virus-specific host immune response, including cytotoxic HBV-specific CD8^+^ *T* cell and natural killer cell responses, B-cell-mediated humoral immunity, and cytokine-mediated non-cytolytic responses (Lang et al., 2019). Consistent with this, the resolution of HBV infection, with or without the presence of anti-HBs, requires CD4^+^ *T* cells for effective virus-specific CD8^+^ *T* cell responses and B-cell antibody production (Lau et al., 2002). Preclinical models have shown that therapeutic vaccines (inducing CD4^+^and CD8^+^T cell responses) and engineered HBV-specific T cells can reduce HBV viral loads, but these results have not yet been confirmed in humans due to potentially irreversible T-cell defects and the long recovery time (Hoogeveen and Boonstra, 2020; Wedemeyer et al., 2009; Dusheiko et al., 2023). Human studies are underway to evaluate the efficacy of ICIs in combination with therapeutic vaccines, particularly in patients with chronic HBV infection, to achieve an HBV cure (Gane et al., 2019; Feld et al., 2023; Cargill and Barnes, 2021). Although a limited number of trials have achieved HBsAg loss, combining therapeutic vaccines with antivirals that have a high genetic barrier to resistance is also a promising approach for achieving a complete cure (Dusheiko et al., 2023; Feld et al., 2023).

However, given the complex defense mechanisms established by the HBV, monotherapies are unlikely to achieve a cure. Consequently, combination therapy has emerged as the central direction in current clinical research for chronic hepatitis B. This strategy typically follows three interconnected and sequential phases: the initial phase involves potent suppression of viral replication to alleviate the burden on the immune system; the core phase focuses on substantially reducing the viral antigen load to break the high antigen-induced immune paralysis; and the final phase aims to activate virus-specific immune responses, enabling the immune system to clear HBV-infected hepatocytes. These three phases form a coherent logical progression from "viral control" to "immune disinhibition" and finally to "viral clearance," representing the most promising pathway towards achieving functional cure for chronic hepatitis B (Feld et al., 2023; Wong et al., 2025).

## 6 An integrated clinical decision-making flowchart

To harmonize divergent recommendations across major guidelines, our flowchart adopts a unified approach. For HBsAg-positive patients, we consistently recommend immediate prophylactic antiviral therapy, reflecting universal consensus. In HBsAg-negative/anti-HBc-positive cases, we introduce risk stratification: high-risk immunosuppressive regimens trigger prophylactic therapy (aligning with EASL and select AASLD guidelines), while moderate/low-risk scenarios adopt monitored preemptive strategies (per APASL and partial AASLD recommendations). To resolve discordant HBV DNA thresholds for reactivation, a two-step verification algorithm is proposed: an initial sensitivity-oriented phase (DNA increase >1–2 log10 IU/mL) flags "suspected early reactivation," followed by a specificity-driven phase (absolute DNA ≥2000 IU/mL) to confirm diagnosis. This dual-threshold model integrates AASLD/EASL's emphasis on early dynamics with APASL's absolute cutoffs, ensuring comprehensive detection while maintaining diagnostic precision.

Fig. 2

**Fig. 2:**
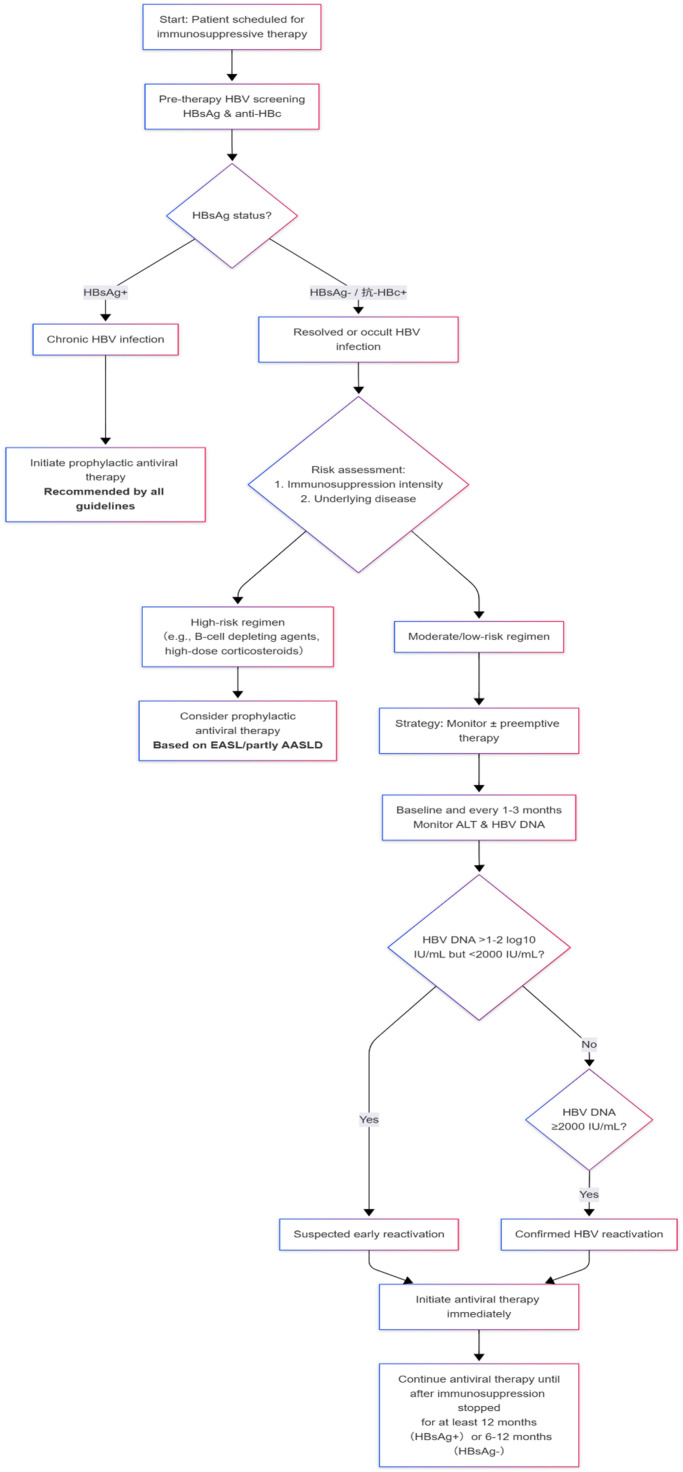
An Integrated Clinical decision-making flowchart.

## 7 Summary and future perspectives

Unlike traditional immunosuppressive therapies, ICIs enhance immune responses by blocking T-cell inhibitory signals. This mechanism may disrupt the immune tolerance to HBV, leading to viral replication reactivation. This unique pathophysiological process necessitates a re-evaluation of HBV screening strategies in clinical practice. Dynamic risk assessments based on viral load, serological markers, and immune function status should be implemented for patients undergoing ICIs therapy, thereby ensuring antitumor efficacy while effectively managing HBV reactivation.

Based on the above summary, we believe that there are several aspects that need to be made efforts to effectively control hepatitis B reactivation in clinical practice:

1. Research on the mechanisms underlying ICI-induced HBV reactivation has advanced significantly in recent years. However, most studies have focused on genetic correlations with clinical outcomes, and no definitive key protein has been identified. Further elucidation of critical rate-limiting or regulatory steps in the HBV reactivation signaling pathway is essential. This knowledge would facilitate the identification of predictive biomarkers for assessing HBV reactivation risk in clinical settings and contribute to the development of novel antiviral therapies.
2. Early hepatitis B virus prevention and antiviral therapy are of great value for ICI treatment, but the risk of side effects from the use of nucleoside analogue therapy should be paid attention to.
3. Multiple global research institutions (including American Society of Clinical Oncology, ASCO; European Association for the Study of the Liver, EASL; American College of Gastroenterology, ACG; National Comprehensive Cancer Network, NCCL; Chinese Medical Association, CMA; China Anti-Cancer Association, CACA) recommend that all cancer patients or patients using potent immunosuppressants should be screened for HBV before immunotherapy, and patients who are positive for HBsAg or anti-HBc should receive prophylactic antiviral therapy. It is worth noting that patients with occult hepatitis B virus infection (OBI) with Anti-HBc negative still have the risk of reactivation of hepatitis B virus.

In addition, there are areas that need to be strengthened beyond clinical work:

1. The dissemination of popular science knowledge related to hepatitis B virus and the popularization of vaccination need to be further strengthened.
2. The basic and clinical research on reactivation of hepatitis B virus needs to be further strengthened, and the investment in funds needs to be further increased. The necessity and importance of this research should not be ignored because of the proposal of the goal of effectively controlling hepatitis B by 2030.
3. Establishing a public database for patients experiencing HBV reactivation would enhance clinical practice and facilitate research on disease background, treatment approaches, and management strategies.

Infection with HBV is highly prevalent worldwide. Despite immune control, HBV can persist within the nucleus of hepatocytes as cccDNA, which has a potential to reactivate spontaneously or under certain conditions. In particular, the risk of HBV reactivation increases in patients receiving ICIs treatment. While the precise mechanisms of ICI-induced HBV reactivation remain unclear, it can be effectively managed through early antiviral treatment in patients with prior HBV infection and prophylactic antiviral therapy in those with chronic HBV infection. Therefore, testing and monitoring HBV serology are recommended for patients who are eligible for ICIs treatment, and prompt initiation of antiviral therapy is necessary when HBV reactivation is detected.

## CRediT authorship contribution statement

**Huajie Xie:** Writing – original draft, Visualization, Supervision, Resources, Methodology, Investigation. **Meijun Lv:** Resources, Investigation, Data curation. **Qin Jia:** Resources, Investigation, Data curation. **Yanyan Wang:** Resources, Investigation. **Wanlin Na:** Validation, Methodology, Investigation. **Yuan Liu:** Validation, Methodology, Investigation. **Kai Chang:** Writing – review & editing, Funding acquisition, Conceptualization.

## Declaration of competing interest

The authors declare that they have no known competing financial interests or personal relationships that could have appeared to influence the work reported in this paper.

## Data Availability

Data will be made available on request.
